# Serpentine supravenous hyperpigmentation induced by chemotherapy: a systematic review

**DOI:** 10.1007/s00403-024-03057-2

**Published:** 2024-05-24

**Authors:** Judy Shan, Bianca C. Obiakor, Justin Cheng, Rony A. Francois, Allison S. Dobry

**Affiliations:** 1 School of Medicine, University of California, San Francisco, San Francisco, CA USA; 2 Keck School of Medicine, University of Southern California, Los Angeles, CA USA; 3 Department of Dermatology, University of California, San Francisco, 1701 Divisadero, San Francisco, CA 94115 USA

**Keywords:** Supravenous serpentine hyperpigmentation, Chemotherapy, Dermatooncology, Skin hyperpigmentation, Infusion reaction

## Abstract

Serpentine supravenous hyperpigmentation (SSH) describes increased skin pigmentation that develops in the area immediately overlying the vessels through which chemotherapeutic drugs are administered. While SSH can be cosmetically distressing and there are no definitive management options, the literature is severely limited and the variations in clinical presentation, risk factors, and histopathology of SSH across patients are not well understood. We aimed to systematically summarize characteristics from current available data, and thus improve SSH awareness and management. A literature search was conducted in PubMed using specific eligibility criteria through the end of December 2022. Included articles focused on patients who experienced SSH after chemotherapy infusion. Study quality was assessed using a modified Oxford Centre for Evidence-Based Medicine quality rating scheme. Of the 41 articles identified by literature search, 24 met eligibility criteria. Two additional articles were identified through the reference sections of retrieved articles, for 26 articles total. All articles were case reports, representing 28 patients total. Locations of SSH were mostly in the forearm near the site of injection (85%), and the most common associated symptom was erythema. Histopathologic analysis was available for half of cases, the majority of which were inflammatory in nature. The most common inflammatory pattern observed was a vacuolar/lichenoid interface dermatitis. Duration of SSH ranged from days to > 1 year after the chemotherapy was stopped. Six (21%) patients were managed with topical steroids and oral vasodilators, six (21%) patients switched to central venous infusion rather than peripheral infusion, five (18%) patients received only supportive care, three (11%) patients received venous washing with chemotherapy, three (11%) patients stopped chemotherapy, and one (4%) patient reduced the chemotherapy dosage. Ten (36%) patients attained complete resolution, seven (25%) had SSH that was near resolution/fading, and three (11%) had persistent hyperpigmentation. Although SSH often spontaneously resolves once the chemotherapeutic agent is stopped, it can persist in some patients and cause significant distress. As the literature is severely limited and there are no definitive treatments, additional research using more standardized definitions and methods of assessments is necessary to improve characterization of SSH and evaluate potential interventions.

## Introduction

Serpentine supravenous hyperpigmentation (SSH) is a term used to describe a phenomenon in which increased skin pigmentation develops in the area immediately overlying the vessels through which chemotherapeutic drugs are administered. SSH is reported in 2–5% of patients, and data characterizing its clinical and histopathological course remains scarce [1]. SSH can be cosmetically distressing (especially in patients with skin of color who may have more prolonged pigment alteration) and there are no definitive management or treatment options [2]. As the literature is limited largely to case reports and the true incidence of cases is likely underreported, it is difficult to fully understand the variations in clinical presentation, risk factors, and histopathologic spectrum of SSH across patients. Our review aims to address this gap in the literature by summarizing characteristics from the current available data, and thus better inform future awareness and management of this condition.

## Methods

### Search strategy

Two reviewers (J.S. and J.C.) performed a literature search through the PubMed database using a combination of the key words "supravenous hyperpigmentation”, “chemotherapy”, “serpentine”, “supravenous dermatitis” and “supravenous eruption” for articles that were published through the end of December 2022.

### Study selection

Three reviewers (J.S., J.C., and B.O.) screened titles and abstracts of all references and then full texts as indicated. We also identified additional relevant studies from the reference sections of included articles. We included reports of patients who experienced skin color changes or reactions over their blood vessels after administration of chemotherapy. We then excluded studies of the wrong study design (e.g., reviews, editorials), studies that were not focused on SSH reactions due to chemotherapy, and studies that were not accessible through our affiliated institutions.

### Data collection and review

Reviewers abstracted data from the selected studies using a structured computerized data collection instrument. Variables of interest included cancer type, drug class and name, drug reaction morphology/onset/location/duration, histological findings and drug reaction management/outcomes.

### Quality assessment

The quality rating scheme used was modified from the Oxford Centre for Evidence-Based Medicine for ratings of individual studies: (1) properly powered and conducted randomized clinical trial or systematic review with meta-analysis, (2) well-designed controlled trial without randomization or prospective comparative cohort trial, (3) case–control study or retrospective cohort study, (4) case series with or without intervention or cross-sectional study, and (5) opinion of respected authorities or case reports. The quality assessment was performed by two authors (J.S. and J.C.).

## Results

**Fig. 1:**
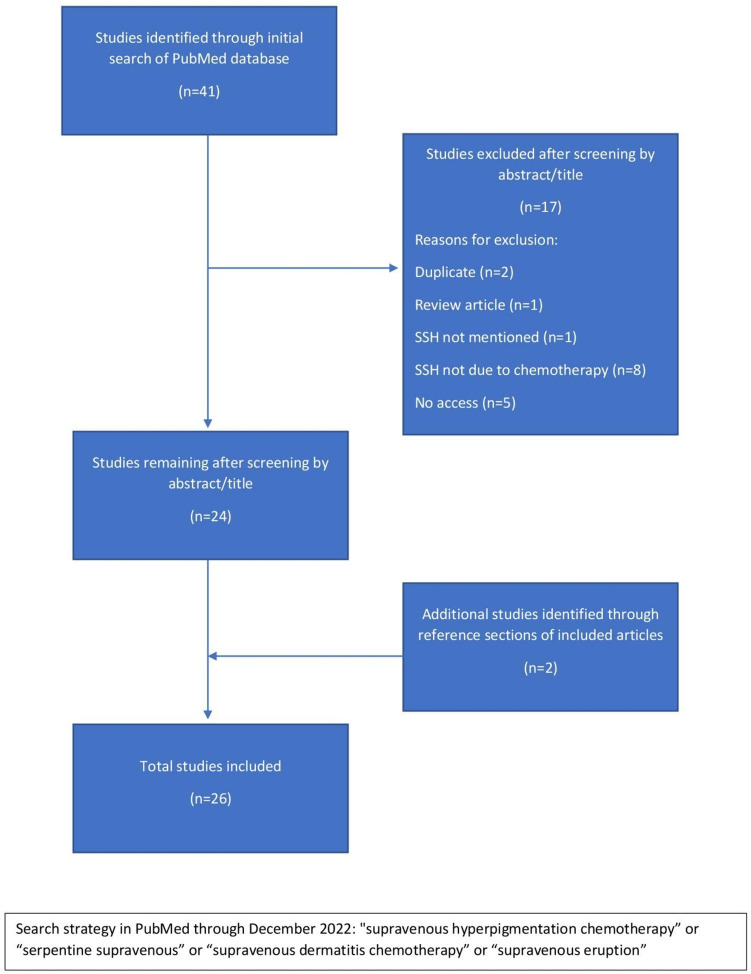
PRISMA Flow Diagram of Selected Studies

Of the 41 articles identified by literature search, 24 met eligibility criteria. Two additional articles were identified through the reference sections of retrieved articles (Fig. 1). We included 26 articles in total, all of which were case reports and had a quality score of 5 according to the modified quality rating scheme from the Oxford Centre for Evidence-Based Medicine for ratings of individual studies. There were 28 patients total, with 9 (32%) female and 19 (68%) male (Table 1). The mean age of onset was 46 years old, and patient age ranged from 7 to 72 years old. Cancer types included breast (8), gastrointestinal (8), hematologic (5), lung (4), sarcomatous (2), and prostate (1). Locations of SSH were mostly in the forearm near the site of injection (85%), although two cases reported SSH on the trunk. Onset of a supravenous cutaneous drug reaction ranged from thirty minutes up to six months after the initial infusion. Duration of SSH ranged from a few days to greater than a year after the chemotherapy was stopped.

**Table 1:** Management and Outcomes of Serpentine Supravenous Hyperpigmentation

Patient age/sex	Cancer category	Classes of oncologic agent	Location	Onset	Duration	Initial Inflammatory reaction or stage?	Management or treatment	Was chemotherapy stopped?	Outcomes
45F [5]	Breast	Antimetabolite	NR	NR	NR	NR	Stopped chemo	yes	Complete resolution
30F [13]	Breast	Taxane	Upper extremity	1 week	NR	No	Stopped chemo	yes	Complete resolution
7M [14]	Hematologic	Antimetabolite, anthracycline	Trunk	1 month	NR	No	NR	NR	NR
15M [14]	Hematologic	Anthracycline, antitumor antibiotic, vinca alkaloid, alkylating agent	Upper extremity	6 months	NR	No	NR	NR	NR
52M [4]	Lung	Alkylating agent, vinca alkaloid	Upper extremity	2 weeks	Few days	Yes	Topical steroids, boric acid dressings, oral NSAIDs	NR	Persistent hyperpigmentation
54F [15]	Breast	Alkylating agent, anthracycline, antimetabolite	Upper extremity	6 weeks	3 months after chemotherapy completion	No	Pentoxifylline	No	Persistent sclerosis, resolution of hyperpigmentation
50M [16]	Hematologic	Alkylating agent, anthracycline, corticosteroid, monoclonal antibody, vinca alkaloid	Upper extremity	10 days	4 months	Yes	Supportive care	No	Near resolution
72M [17]	Breast	Taxane, anthracycline, alkylating agent	Upper and lower extremity	2 days	NR	yes	Topical steroids and emollients	NR	NR
15M [18]	Hematologic	Anthracycline, vinca alkaloid, topoisomerase inhibitor	Upper Extremity	4 weeks	> yr after chemotherapy completion	No	Nothing	No	Faded but persistent hyperpigmentation
51F [19]	Breast	Vinca alkaloid	Upper extremity	30 min	NR	No	Nothing	No	Complete resolution
60M [20]	Lung	Vinca alkaloid	Upper extremity	24 days	NR	No	NR	NR	NR
15M [21]	Sarcoma	Vinca alkaloid, antitumor antibiotic	Upper extremity	6 h	NR	Yes	Venous washing	No	NR
44M [1]	GI	Antimetabolite, alkylating agent	Upper extremity	6 days	4 weeks	Yes	Central infusion	No	Persistent hyperpigmentation
42F [7]	GI	Antimetabolite, alkylating agent	Upper extremity	10 days	4 months	Yes	Central infusion, topical steroids	No	Faded but persistent hyperpigmentation
45F [3]	Breast	Taxane	Upper extremity	1 day	1 week	Yes	Topical steroids	no	Faded but persistent hyperpigmentation
61M [22]	GI	Antimetabolite, alkylating agent	Upper extremity	NR	8 weeks	yes	Central infusion	NR	Complete Resolution
56M [8]	Prostate	Antimetabolite	Upper extremity	24 weeks	NR	No	NR	NR	NR
46M [12]	Lung	Taxane	Upper extremity	2 days	24 weeks	Yes	Venous washing	No	Complete resolution
30M [23]	GI	Antimetabolite	Upper extremity	2 cycles	NR	No	Nothing	No	NR
38M [9]	GI	Antimetabolite, corticosteroid, alkylating agent	Upper extremity	8 weeks	4 weeks	No	Central infusion	No	Faded but persistent hyperpigmentation
52M [24]	GI	Taxane	Upper extremity	4 weeks	2 months	No	Nothing	No	Faded but persistent hyperpigmentation
58F [25]	Sarcoma	Antimetabolite, taxane	Upper Extremity	2 weeks	2 months	No	Central infusion	No	Complete resolution
47M [6]	GI	Antimetabolite, alkylating agent, corticosteroid	Upper extremity	4 days	3 months	Yes	Stopped chemo	Yes	Complete resolution
58M [26]	Hematologic	Proteasome inhibitor	Upper extremity	8 h	Several months	Yes	Venous washing	No	Persistent hyperpigmentation
68F [27]	Breast	Taxane	Upper extremity	Few days after second cycle	7 days	Yes	Topical steroids	No	Persistent hyperpigmentation
65F [27]	Breast	Taxane	Upper extremity	few days after third cycle	3 months	Yes	Topical steroids	No	Complete resolution
69M [28]	GI	Antimetabolite	Trunk	2 days	7 days	Yes	Reduced chemo	No	Faded but persistent hyperpigmentation
52M [11]	Lung	Alkylating agent, antimetabolite	Upper extremity	2 cycles	NR	No	Central infusion	No	NR

Missing data were as follows: onset (7.1%), duration (39.3%), management (21.4%), and outcomes (28.6%)

*NR* not reported

Of the 27 patients with documented morphology findings and symptom course, 14 (52%) experienced an initial inflammatory reaction (i.e., erythema, edema, fever, chills) prior to the onset of hyperpigmentation. The most commonly reported inflammatory symptom was erythema (86%).

Of the 28 reported patients, 13 (46%) patients had biopsy specimens with documented histopathology (Table 2). Nine of the 13 specimens (69%) demonstrated an inflammatory pattern, two (15%) revealed a pauci-inflammatory pattern, and two (15%) had a non-inflammatory pattern. The vast majority of the inflammatory specimens (78%) demonstrated a vacuolar or lichenoid interface dermatitis (either as a primary or mixed pattern) of varying intensity, while the remaining two inflammatory specimens demonstrated a perivascular/interstitial pattern of lymphocytic inflammation. The two pauci-inflammatory patterns demonstrated variable fibrosis, with one specimen having epidermal atrophy and papillary dermal fibrosis, and the other specimen with extensive dermal and deep vessel sclerosis as well as perivascular fibrosis. Regarding the type of pigmentation, most specimens (69%) demonstrated increased melanin pigment, with two specimens (15%) having extravasated erythrocytes, and two specimens 15% having no identifiable pigment deposition.

**Table 2:** Histopathologic Findings of Serpentine Supravenous Hyperpigmentation

Patient age/sex	Infiltrate: Inflammatory vs. sparse/none	Infiltrate pattern	Infiltrate cell type	Type of pigmentation/discoloration	Pigmentation location
52M [4]	Inflammatory	Perivascular and interstitial	Lymphocytic	Extravasated erythrocytes	Perivascular
54F [15]	Pauci-inflammatory	Fibrotic/sclerotic	Fibroblasts	None	None
72M [17]	Inflammatory	Lichenoid interface and perivascular	Mononuclear	Melanin	Epidermal
15M [18]	Inflammatory	NR	Lymphocytic	Melanin	Epidermal and superficial dermal
60M [20]	Non-inflammatory	N/A	N/A	Melanin	Mixed: epidermal and superficial dermal
15M [21]	Inflammatory	Vacuolar interface, cytotoxic/perieccrine	Mixed: lymphocytic, histiocytic and polymorphonuclear	Melanin	Mixed: epidermal and superficial dermal
42F [7]	Pauci-inflammatory	Perivascular	Lymphocytic	Melanin	Superficial perivascular
45F [3]	Inflammatory	Mixed spongiotic and lichenoid interface dermatitis	Mixed: lymphocytic and mononuclear	None	N/A
46M [12]	Inflammatory	Vacuolar interface and perivascular	Lymphoplasmacytic	Melanin	Epidermal
30M [23]	Non-inflammatory	N/A	N/A	Melanin	Mixed: epidermal and superficial dermal
47M [6]	Inflammatory	Vacuolar interface dermatitis	Lymphocytic	Melanin	Superficial dermal
58M [26]	Inflammatory	Cell poor interface dermatitis and perivascular	Lymphocytic	Extravasated erythrocytes	Perivascular
69M [28]	Inflammatory	Superficial perivascular	Lymphocytic	Melanin	Epidermal

Missing data were as follows: histologic findings (53.6%)

*N/A* not applicable, *NR* not reported

Chemotherapy was not stopped after the onset of SSH in 17 (63%) patients. Of these patients, six (35%) attained complete resolution of their SSH, six (35%) had SSH that were near resolution or were in the process of fading, and three (18%) had persistent hyperpigmentation. Long-term outcomes were not available for two of these patients.

Regarding management, six (21%) patients were managed with topical steroids and oral vasodilators, six (21%) patients switched their chemotherapy route to central venous infusion rather than peripheral infusion, five (18%) patients were simply observed or were provided supportive care, three (11%) patients received venous washing with their chemotherapy, three (11%) patients stopped chemotherapy, and one (4%) patient reduced the dose of chemotherapy.

In terms of overall outcome, ten (36%) patients attained complete resolution of their SSH, seven (25%) had SSH that was near resolution or in the process of fading, and three (11%) had persistent hyperpigmentation.

## Discussion

Serpentine supravenous hyperpigmentation describes increased skin pigmentation overlying the superficial vessel network through which chemotherapy is administered. This morphological pattern has also been described as a supravenous hyperpigmented eruption. The clinical presentation is variable; in some it may initially appear as erythematous streaks located immediately over the injected veins, and subsequently develop into hyperpigmented linear patches over the course of days to weeks [3]. Other patients may only experience late pigmentary changes that develop weeks or months into their treatment, without the initial inflammatory stage, which is also known as supravenous erythematous eruption.

SSH is most commonly associated with chemotherapy infusions, and the single drug that is most commonly associated with SSH in the literature is the antimetabolite 5-fluorouracil [4]. Other chemotherapeutic agents that have been associated with SSH are taxanes (i.e., docetaxel), vinca alkaloids (i.e., vinorelbine, vincristine), alkylating agents (i.e., carboplatin, oxaliplatin, cyclophosphamide), antitumor antibiotics (i.e., actinomycin, bleomycin), anthracyclines (i.e., epirubicin, daunorubicin, doxorubicin), proteasome inhibitors (i.e., bortezomib), or any combination of these drugs [4, 5].

SSH has not been well-characterized histopathologically, which is key to better understanding the underlying pathophysiology. Although cytotoxic damage and hypersensitivity have been known to cause pigmentary changes, there is no clear consensus on the pathophysiology for SSH [6]. It has been posited that a potential mechanism for SSH is a loss of blood vessel endothelial integrity due to the intravenous chemotherapy causing a leakage into the epidermis, causing melanosome destruction and resultant pigment release [7–9]. An alternate theory suggests that altered melanosome packaging might occur as a result of chemotherapy exposure [8]. Yet another hypothesis is that the reduced local environment produced by alkylating chemotherapeutic agents leads to depletion of reduced thioredoxin, which then activates tyrosinase, causing hyperpigmentation [10]. Further hypotheses include hyperthermia-induced changes such as reduced cytokine production and increased stimulation of melanocyte hormone receptors, post-inflammatory hyperpigmentation caused by subclinical phlebitis, and increased blood flow causing increased drug deposition in specific areas [5, 11, 12].

Among patients with documented histopathology, all patients who received a taxane agent presented with inflammatory erythema with secondary change and hyperpigmentation. These patients also had evidence of a lichenoid or vacuolar interface dermatitis. Similarly, the only patient who received actinomycin D also presented in this manner with the same histopathology. Interestingly, the only patient who received a combination of an alkylating agent, an antimetabolite, and an anthracycline experienced severe sclerosis of deep vessels as well as the lesional dermis. In the aforementioned cases, pigmentation was mostly due to melanin from melanophages, with extravasated erythrocytes occurring less commonly.

Given the above associations, we propose an additional mechanism for the pathophysiology of SSH. Initially, intravenous administration of cytotoxic chemotherapy would result in compromised endothelial integrity. This which would initially result in erythema in the short term and extravasation of erythrocytes. Interstitial infiltration of the cytotoxic agent would then cause local tissue destruction of varying intensity, depending on the class and/or combination of agents used. In addition to the localized epidermal keratinocyte necrosis, a subsequent immune-mediated cytotoxic lymphocytic response may also occur. This would result in a concomitant lichenoid/vacuolar interface pattern with melanophages, which would ultimately result in hyperpigmentation.

Some literature suggests that venous washing may help to prevent SSH by reducing venous load toxicity [5, 12]. In our study, most cases of SSH were treated with steroids or vasodilators, normal saline venous washing, or resolved spontaneously after discontinuation of the drug. Some patients were also switched from peripheral venous infusion of chemotherapy to central venous infusion.

Future case reports and studies on SSH can increase awareness and help to improve future management of SSH by reporting more specifically on onset, duration, management, histopathologic findings and outcomes. It would be particularly helpful to more clearly delineate the difference in timing between an initial inflammatory infusion reaction, true SSH, and SSH-like post-inflammatory hyperpigmentation. Once these differences are more clearly elucidated, diagnoses will improve in accuracy and patient expectations may be managed more appropriately.

## Limitations

Our study is limited in that it primarily includes case reports, which may not provide the robust evidence as would be provided by randomized controlled trials and prospective studies. Furthermore, the lack of standardization in data reporting among case reports poses challenges to drawing definitive conclusions from our study. This also resulted in missing data for certain key variables in our study, which may impact the overall analytic completeness of our study. Finally, due to the paucity of available literature on SSH, the number of patients included in our study is relatively small, which limits the generalizability of our study findings.

## Conclusion

Serpentine supravenous hyperpigmentation is a side effect that occurs secondary to chemotherapy infusions. It typically manifests as hyperpigmented patches over the venous network of the arm in which chemotherapy is infused, and can sometimes be preceded by an inflammatory stage with erythema. Oftentimes, SSH is benign and self-limiting, and chemotherapy can be continued. Although the hyperpigmentation often spontaneously resolves once the chemotherapeutic agent is stopped, SSH can persist in some patients and cause distress. Options to prevent SSH include altering infusion protocols either through switching to central venous infusion or performing venous washing with chemotherapy. Alternatively, if SSH does persist, topical corticosteroids may offer some improvement. Given the varied histopathologic findings, biopsy should not be routinely performed on all patients suspected to have SSH. Rather, biopsy should be considered for select cases with unusual clinical features, such as cases that do not respond to the management strategies above. As the literature is severely limited and there are no definitive treatments, additional research using more standardized definitions and methods of assessment is necessary to better characterize SSH and evaluate potential intervention strategies.

## Data Availability

No datasets were generated or analysed during the current study.
